# The Effects of a Multi-Ingredient Performance Supplement Combined with Resistance Training on Exercise Volume, Muscular Strength, and Body Composition

**DOI:** 10.3390/sports7060152

**Published:** 2019-06-25

**Authors:** Dean Directo, Michael W.H. Wong, Marcus L. Elam, Paul Falcone, Adam Osmond, Edward Jo

**Affiliations:** 1 Human Performance Research Laboratory, Department of Kinesiology and Health Promotion, California State University Pomona, Pomona, CA 92805, USA; dean.directo@gmail.com (D.D.); mike.wong203@gmail.com (M.W.H.W.); paul.h.falcone@gmail.com (P.F.); adosmond7@gmail.com (A.O.); 2 Department of Nutrition and Food Science, California State University Pomona, Pomona, CA 92805, USA; mlelam@gmail.com

**Keywords:** creatine, beta-alanine, ergogenic, BCAA, skeletal muscle

## Abstract

The effects of a multi-ingredient performance supplement (MIPS) incorporating a mixture of branched chain amino acids, beta-alanine, glutamine, creatine, and piperine on resistance training (RT)-induced adaptations remains unclear. Therefore, the purpose of this study was to investigate the effects of this investigational MIPS during six weeks of RT on performance and body composition. Thirty recreationally trained males and females were recruited for this pair-matched, double-blind, placebo-controlled investigation. Subjects were assigned to consume either an experimental MIPS (MIPS) (*n* = 15) or a placebo (PLA) (*n* = 15) concurrently with a six-week periodized RT program. Body composition, one-repetition maximum (1RM), and muscular power were assessed at pre- and post-training. Weekly relative volume load was compared between groups. The MIPS and PLA groups demonstrated a significant increase in total body mass (MIPS = +2.9 ± 1.3%; PLA = +2.5 ± 1.7%) and lean mass (MIPS = +5.0 ± 2.1%; PLA = +3.1 ± 1.9%) (*p* < 0.001) with no changes in fat mass. There were no group × time interactions for any of the body composition measures. Both groups demonstrated similar improvements in maximum strength for the back squat, bench press, and deadlift as well as lower body power from pre- to post-training (*p* < 0.001). Within the limitations of the current investigation, results failed to demonstrate the benefits of the experimental MIPS for muscular strength and body composition across six weeks of RT compared to PLA.

## 1. Introduction

A relatively large body of research has been dedicated to investigating methods of potentiating exercise performance and training-induced adaptations. A widely exploited strategy is the use of dietary supplements containing bioactive compounds or nutrients designed specifically to promote an ergogenic response or support metabolic demands during exercise [1]. Concurrent supplementation of dietary performance supplements during resistance training (RT) has gained widespread popularity among competitive and recreational athletes alike due to their purported function of improving exercise quality and augmenting the rate of training adaptations [2,3,4]. An increasingly popular method of supplementation involves consumption of a single mixture of various substances, commonly referred to as a multi-ingredient performance supplement (MIPS). Although MIPS vary widely in ingredient composition, a recent product includes a blend of creatine, beta-alanine, branched-chain amino acids (BCAA), glutamine, and plant-based compounds such as piperine [5,6,7,8].

A large proportion of currently available MIPS compounds are implicated as “pre-workout” supplements with the intent of inducing a subsequent acute ergogenic effect via stimulatory ingredients such as caffeine. However, an alternate class of MIPS are designed with the aim of providing metabolic support through nutritional substrate provision for bioenergetic or anabolic processes (i.e., myofibrillar protein synthesis) as well as promotion of skeletal muscle recovery. Regardless of the ongoing controversy of the ideal type of supplements or nutrients for performance enhancement, BCAA, beta-alanine, glutamine, creatine, and piperine have demonstrated a variable degree of ergogenic effects on the human body when consumed individually [9,10,11,12,13]. This has stimulated the adoption of MIPS that incorporate a blend of these substances in efforts to combine the ergogenic properties of each ingredient into one efficient supplement. The multi-ingredient approach to ergogenic supplementation has previously shown to facilitate the physiological adaptations associated with RT [14,15,16]. However, there is currently limited evidence regarding the efficacy of a MIPS comprised specifically of a mixture of BCAA, creatine, beta-alanine, glutamine, and black pepper fruit extract (piperine) on morphological and functional RT-induced muscular adaptations. Therefore, the purpose of this study was to investigate the effects of a MIPS comprised of the aforementioned ingredients on exercise volume, muscular function, and body composition across six weeks of resistance training.

## 2. Materials and Methods

### 2.1. Experimental Approach to the Problem

**Figure 1:**
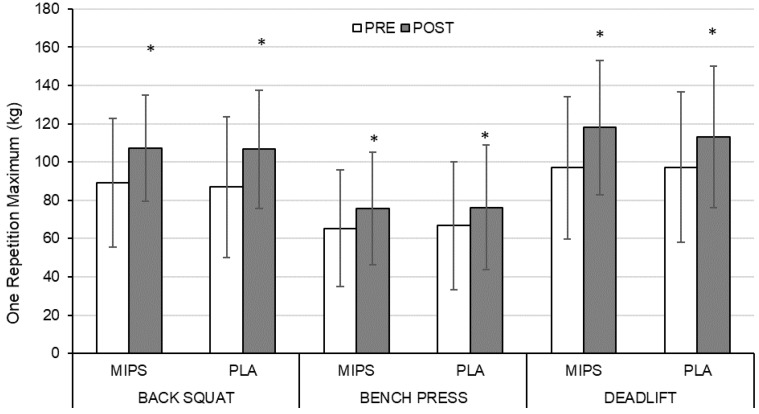
Pre- to post-training relative change in absolute one repetition maximum for back squat, bench press, and deadlift for MIPS and PLA. MIPS = experimental MIPS group, PLA = placebo group. Data presented as mean ± SD. * Significant pre- to post-training change (*p* < 0.001).

A double-blind, placebo-controlled experiment with a mixed between- (treatment group) and within-groups (time) factor design was implemented for this study (Figure 1). Subjects were pair-matched by relative strength and sex and placed into one of two treatment groups for a total of eight weeks with six weeks of RT: experimental MIPS group (MIPS, *n* = 15) or placebo control group (PLA, *n* = 15). Baseline descriptive measures are presented in Table 1. The first week of the experiment incorporated familiarization of performance testing protocols on Monday, baseline assessment of body composition and strength on Wednesday, and baseline assessment of muscular power followed by familiarization of resistance exercises on Friday. For next six weeks, both groups performed RT as detailed below. Concurrently with RT, the MIPS group consumed a multi-ingredient dietary supplement containing a mixture of BCAA, beta-alanine, creatine hydrochloride, piperine, and glutamine while PLA consumed an isocaloric and taste-matched placebo (details below). During the week after the completion of the RT protocol, subjects underwent post-treatment testing procedures. This study was approved by the Institutional Review Board (IRB#15-0156).

### 2.2. Subjects

Thirty (*n* = 30) healthy college-aged, resistance-trained male (*n* = 14) and female (*n* = 16) subjects were recruited for this study. Prior to participation, each subject signed a document of informed consent. Subjects met the following inclusion criteria: (1) age = 18 to 32 years, (2) not a competitive athlete in a collegiate or professional sport, and (3) recreationally resistance-trained as defined by resistance exercise performed 3–4 days/week for six months prior to the start of the study and could perform the back squat and bench press with a load 1.0 (male) or 0.75 (female) and 0.75 (male) or 0.5 (female) times their bodyweight, respectively. Subjects were excluded from participation if they reported a medical or surgical history that would have contraindicated the experimental protocol and/or confounded the interpretation of results. In addition, daily use of any dietary supplements within 12 weeks prior to the study called for exclusion.

### 2.3. Resistance Training Protocol

The RT protocol comprised of three training sessions per week on nonconsecutive days. RT was performed for six weeks following an initial week in which familiarization of the exercises was implemented. During the six-week RT period, Session 1 of each week incorporated the following exercises in the listed order: back squat, bench press, deadlifts, shoulder press, rows, and dumbbell chest press. The back squat, bench press, and deadlift were performed for four sets for 12 repetitions, while the remaining exercises were performed for three sets at 10 repetitions with 10-repetition maximum (RM) loads. Session 2 of each week incorporated the same exercises as Session 1 but excluded the deadlifts. The back squat and bench press were performed for four sets for 10 repetitions while the remaining exercises were performed for three sets for eight repetitions with 8RM loads. Session 3 of each week consisted of the same exercises as Session 1 in the same order. The back squat, bench press, and deadlift were prescribed at five sets for eight repetitions while remaining exercises were prescribed at three sets for six repetitions with 6RM loads. The initial 10RM loads were determined during the familiarization session in the week before the six-week RT by attempting 10 repetitions with an estimated 10RM and adjusting the load in subsequent attempts based on rating of perceived exertion as presented previously [17]. Initial 8RM and 6RM loads were estimated based on the 10RM load (8RM = 10 lbs more than 10RM and 6RM = 20 lbs more than 10RM). For the initial training week, a 1RM-based load prescription was implemented for the back squat, deadlift, and bench press (Session 1 = 60% 1RM, Session 2 = 65% 1RM, and Session 3 = 70% 1RM). Thereafter, training load adjustments were made on a set-to-set and week-to-week basis as described previously [17,18]. The final week of training incorporated a taper in which volume was reduced. A certified strength and conditioning specialist monitored all training sessions.

### 2.4. Dietary Supplementation Protocols

The experimental dietary supplement was a proprietary multi-ingredient compound comprised of a blend of BCAA (6 g/serving, 4:1:1 leucine, isoleucine, and valine ratio), L-glutamine (3 g/serving), beta-alanine (2 g/serving), creatine HCl (2 g/serving), and piperine (5 mg/serving) (MusclePharm Co., Denver, CO, USA). A single serving provided 1 g of carbohydrate constituting approximately 5 kcal. To test the experimental MIPS under manufacturer dosing recommendations, subjects consumed one serving of their assigned supplement immediately post-workout and before sleep on training days (Monday, Wednesday, and Friday); two separate single servings were taken intersession upon waking and before sleep on Tuesday, Thursday, Saturday, and Sunday. The placebo was a calorie- and taste-matched supplement without the active ingredients listed above, and the same dosing instructions as MIPS were given. The MIPS and placebo were administered in a double-blind manner. Containers for each supplement were labeled “A” or “B”. Information regarding the content of supplement “A” and “B” was documented by the manufacturer and sealed in an envelope until completion of data analysis.

### 2.5. Muscular Strength Testing

Baseline and post-treatment 1RM strength tests were administered for the back squat, bench press, and deadlift exercises in the listed order. Prior to testing, subjects completed a standardized dynamic warm-up lasting approximately 10 min. Afterwards, the 1RM test was administered according to procedures previously described by Zourdos et al. [17]. A RT-specific rating of perceived exertion based upon repetitions-in-reserve was used to aid in load selection with successive attempts [17]. A successful attempt was defined according to the standards presented by the International Powerlifting Federation Technical Rulebook [19]. A certified strength and conditioning specialist administered all 1RM tests.

### 2.6. Muscular Power Testing

Muscular power during the back squat was determined utilizing a linear position transducer (Tendo Power Analyzer, Tendo Sports Machines, Trencin, Slovakia) [20]. The free end of the cable was tethered to the end of the barbell while the transducer was secured to the floor. Subjects performed three 4-repetition trials against a load commensurate to 70% and 80% of the subject’s baseline 1RM. The trial with the greatest average power across the four repetitions was used for analysis. Subjects were instructed to perform each trial with maximum effort during the concentric phase. Depth of the descent was controlled using an adjustable seat, and eccentric tempo was set at 2 s. Peak power (PWR_PEAK_) and average power (PWR_AVG_) were deduced from force, barbell displacement, and time of barbell displacement data using data acquisition software interfaced to the device [Intraclass Correlation Coefficient (3,1) = 0.89, 95%CI = 0.82–0.93]. Assessment of power was administered 48 h following the pre- and post-training maximum strength tests.

### 2.7. Body Composition Testing

Body composition was measured by dual-energy X-ray absorptiometry (DXA) (Hologic Discovery-QDR Series Densitometer, Bedford, MA, USA). Fat mass (FM) and non-bone lean mass (LM) (i.e., lean mass – bone mineral content) was analyzed for the whole body. All subjects prior to each scan were free from metallic clothing and accessories. Subjects were tested following first morning void for pre- and post-DXA scans in a fasted state with no food or fluid intake and heavy physical activity prior to the scan. All DXA scans were performed prior to strength tests. The DXA machine was calibrated before each scan using a manufacturer-provided phantom. All DXA measurements and analyses was conducted by a single certified technologist.

### 2.8. Dietary Control

Participants were instructed to avoid any other dietary supplements or ergogenic aids and maintain consistent dietary intake for the duration of the study. Daily dietary intake was monitored using MyFitnessPal (Under Armour Inc., Baltimore, MD, USA). Dietary intake was assessed weekly to ensure that nutrient and caloric intake remained consistent throughout the experimental period.

### 2.9. Statistical Analysis

A 2 (group) × 2 (time) × 2 (sex) analysis of variance (ANOVA) was used for tests of main effects and interactions for all performance and body composition data. In the event of a significant *F*-ratio, a Bonferroni follow-up test was used for pairwise comparisons. Total relative volume load as calculated by multiplying 1RM% load by repetitions were analyzed using an independent *t*-test. All statistical analyses were performed using Statistical Package for Social Science (SPSS, version 14.0 Chicago, IL, USA) with significance set at *p* < 0.05. The effects of treatment were calculated as the percent change in performance measures from baseline to post-treatment between MIPS and PLA. Magnitude-based inference analysis, as described previously [21,22], was used to identify clinically meaningful differences in the delta score of each performance measure between treatment groups. The precision of the magnitude inference was set at 90% confidence limits, using a *p*-value derived from an independent *t*-test comparing mean differences in percent delta change of measures between groups. Threshold values were standard deviations of control group values multiplied by 0.2. Inferences of true differences between PLA and MIPS were determined as beneficial (positive), trivial, or harmful (negative) [22]. Inferences were based on the confidence limit relative to the smallest clinically meaningful effect to be positive, trivial, or negative. Unclear results were reported if the observed confidence interval overlapped both positive and negative values. The probability of the effect was evaluated according to the following scale: <0.5%, most unlikely; 0.5–5%, very unlikely; 5–25%, unlikely; 25–75%, possibly; 75–95%, likely; 95–99.5%, very likely; >99.5%, most likely [21].

## 3. Results

### 3.1. Descriptive Measures

All baseline descriptive measures are indicated in Table 1. There were no significant differences between MIPS and PLA for any baseline descriptive measures.

### 3.2. Body Composition

Pre- and post-training body composition data and respective *p*-values for main time effects are displayed in Table 2. Both MIPS and PLA groups exhibited a significant increase in total body mass (TBM) and LM (*p* < 0.001) with no group × time interaction for these variables. There were no main time effects or group × time interaction for FM. No interaction of sex was detected.

### 3.3. Absolute and Relative Maximum Strength

**Figure 2:**
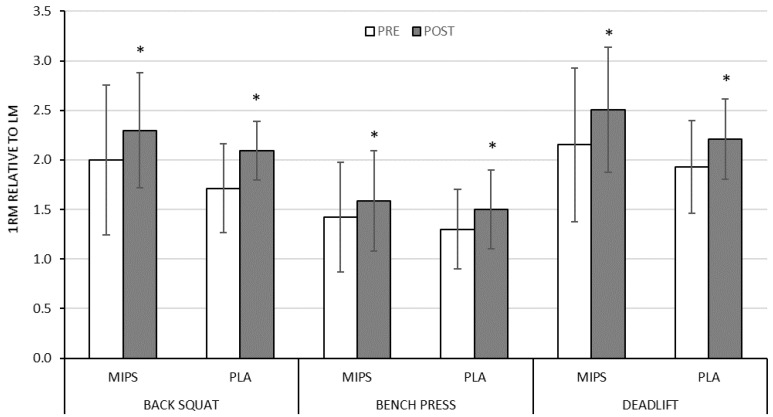
Pre- to post-training relative change in one repetition maximum for back squat, bench press, and deadlift normalized to lean mass for MIPS and PLA. MIPS = experimental MIPS group, PLA = placebo group. Data presented as mean ± SD. * Significant pre- to post-training change (*p* < 0.001).

There was a main time effect indicating a significant 1RM improvement for each exercise for MIPS (back squat = +26.8 ± 23.3%, *p* < 0.001; bench press = +20.5 ± 13.5%, *p* < 0.001; deadlift = +27.6 ± 20.4%, *p* < 0.001) and PLA (back squat = +31.4 ± 24.7%, *p* < 0.001; bench press = +14.3 ± 12.0%, *p* < 0.001; deadlift = +20.4 ± 14.9%, *p* < 0.001) (Figure 1). There was no group × time interaction for back squat, bench press, or deadlift 1RM. Similar statistical outcomes were found for all 1RM measures normalized to LM (Figure 2). No interaction of sex was found.

Magnitude-based inference analysis indicated that the MIPS treatment is “possibly beneficial” in comparison to PLA treatment with regards to percent delta change in absolute and normalized (to LM) bench press and deadlift strength (Table 3). There were unclear results and therefore uncertain qualitative inferences for the difference in percent delta back squat (absolute and normalized to LM).

### 3.4. Lower Body Power

There was no group × time interaction for lower and upper body peak or average power with loads corresponding to 70% or 80% 1RM (Table 4). There was a main time effect in which post-hoc tests showed significant improvements in each measure of lower body power in both MIPS and PLA. There was no interaction of sex.

### 3.5. Relative Volume Load

**Figure 3:**
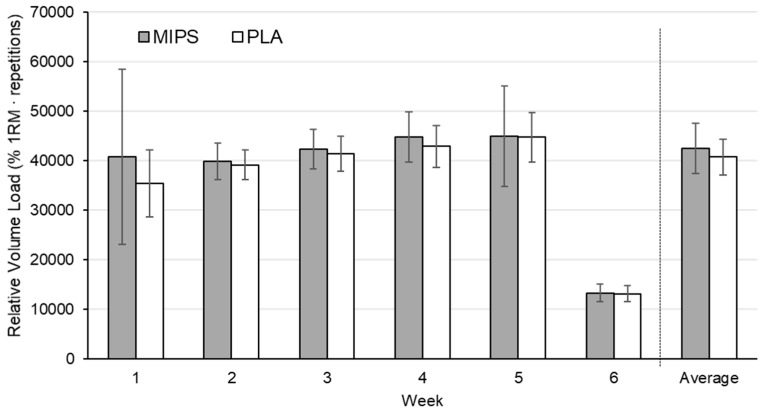
Weekly and average relative volume load between MIPS and PLA. MIPS = experimental MIPS group. PLA = placebo group. Data presented as mean ± SD.

There were no group differences for relative volume load each week and average weekly relative volume load (Figure 3). There was no interaction of sex.

### 3.6. Dietary Intake

Two-way ANOVA (2 groups × 6 weeks) revealed no significant main effect or group × time interactions for average daily caloric, protein, carbohydrate (CHO), or fat intake. Total average daily intake did not differ between groups for caloric (MIPS = 1628.4 ± 159.3 kcals/day vs. PLA = 1708.0 ± 133.8 kcals/day), CHO (MIPS = 175.7 ± 19.3 g/day vs. PLA = 181.1 ± 16.2 g/day), protein (MIPS = 81.7 ± 11.3 g/day vs. PLA = 85.9 ± 9.5 g/day), or fat (MIPS = 57.3 ± 6.4 g/day vs. PLA = 60.2 ± 5.4 g/day) intakes. Within each group, average daily caloric and macronutrient intake per week did not differ among the experimental weeks. There were no interactions of sex.

## 4. Discussion

In summary of the principal findings, daily supplementation of the experimental MIPS failed to elicit any effects on body composition or performance changes across six weeks of RT in comparison to a placebo control. No observable augmentation to weekly and overall training volume was observed with the experimental MIPS treatment, thereby, demonstrating the absence of acute resistance exercise performance benefits. Arguably, the fundamental purpose of MIPS use is to acutely enhance performance such that improved quality of each training bout may ultimately facilitate muscular adaptations. The experimental MIPS, when used as a “post-workout” or “recovery” supplement, is proposed to facilitate these ergogenic effects through increased provision of energy substrates to aid muscle bioenergetics and amino acids to support muscle recovery and protein anabolism. Since the experimental MIPS failed to enhance the quality of each training session as reflected by similar training volume achievements as the placebo group, it would be expected that any RT-induced adaptations would be comparable between treatment groups. The analogous performance gains between treatment groups may be corroborated by the lack of difference in training volume.

The current results regarding training volume are consistent with that of Ormsbee et al. [5] in which authors reported similar outcomes following experimentation with a comparable MIPS mixture. As with the present study, they indicated significant effects of time for upper and lower body strength, however with no group × time interactions, indicating that the experimental MIPS failed to acutely enhance the overall work achieved during each RT bout. Furthermore, Chromiak et al. [23] and Beck et al. [24] arrived at similar conclusions with physically active (1–5 h of weekly strenuous exercise on a regular basis) and untrained males, respectively. In contrast to the afore-mentioned studies, Kreipke et al. [25] found that their experimental MIPS treatment produced significantly greater volume in squat, deadlift, and bench press exercises compared to the placebo group. In addition, Shelmadine et al. [14] and Lowery et al. [26] also reported enhancements in upper body strength with their respective MIPS treatment. However, it is important to note that Lowery et al. [26] employed an eight-week RT program which was two more weeks than that of the present study thereby allowing for an increased window for potential divergences in strength gains to occur. It is also important to emphasize that Shelmadine et al. [14] utilized untrained males while Kreipke et al. [25] and Lowery et al. [26] examined resistance-trained males. Due to the inverse relationship that exists between training status and rate of training progression (i.e., strength increases), there could conceivably be a larger magnitude of effect with untrained subjects [27]. Aside from training status, the composition of MIPS varied across studies with slightly different ingredients and dosages. For example, caffeine, which has previously shown to acutely improve exercise performance, is a notable ingredient absent from the present MIPS but commonly seen in others [1]. Despite the variability across studies regarding MIPS composition, the findings from those studies, in large, point to a key link between training volume and muscular performance enhancement. Specifically, an effective MIPS should fundamentally enhance the quality of each training bout thereby facilitating the rate of training adaptations.

Despite the inclusion of ingredients with some evidential support for ergogenic efficacy, the current MIPS failed to produce a detectable effect on acute performance and RT adaptations. For example, supplementation of beta-alanine in conjunction with RT has shown to attenuate fatigue with consequent increases in training volume compared to a placebo in resistance-trained males [28]. Furthermore, a separate study examining creatine in isolation as well as creatine in combination with beta-alanine found greater strength improvements in both treatment groups compared to a placebo also in resistance-trained males [29]. Despite the inclusion of apparently effective ergogenic ingredients, the nature of our study may not have provided the most favorable conditions for their effects to manifest. For example, a recent position stand on beta-alanine from the International Society of Sports Nutrition suggests that a loading phase of about four weeks is essential for subsequent increase in muscle carnosine levels which is the connecting mechanism between beta-alanine supplementation and performance enhancement [10]. Additionally, a prior review of literature recommends dosages of 2–3 g/day of creatine to increase intramuscular creatine and phosphocreatine stores over a 3–4 weeks period [1]. Thus, it appears that a 3–4-week loading period may be necessary before any effects could occur. Therefore, the length of our experimental timeline may not have provided a sufficient period for ergogenic effects to take place. The results of Lowery et al. [26] seem to support this theory since they observed a positive outcome for upper body strength with their MIPS treatment while utilizing an eight-week RT protocol.

The multi-ingredient approach to performance supplementation certainly presents with limitations in both research and practice. From a research perspective, many studies involving MIPS, such as the present investigation, utilize a pre-engineered blend of ingredients that may or may not in isolation have evidence demonstrating efficacy. In cases even where a MIPS contains evidence-based ingredients, the doses provided in a recommended serving of the supplement may be sub-optimal for a given ingredient and/or individual. This is an inherent limitation in all MIPS research. Moreover, when examining the body of research of single-ingredient performance supplements, it is commonplace to observe relatively large inter-subject variability and a comparable number of positive, negative, and non- responders even in a generally homogenous cohort. This often precludes a statistically detectable effect and keeps the likelihood of even a small worthwhile effect to a minimum. The multi-ingredient approach, even with evidence-supported ingredients, fails to amend these highly variable responses and/or small effects generally associated with dietary supplementation. From a practical application standpoint, athletes or exercising individuals often combine the use of multiple supplements in efforts to acquire a synergistic enhancement of one or more performance attributes. Although synergies between or among single-ingredient supplements are evident, such as between sodium bicarbonate and beta-alanine or caffeine [30,31,32], there are no known data suggesting a potential synergy among any of the ingredients in the present investigational MIPS. Thus, currently there is no clear justification for the application of the current MIPS at least within the setting and population represented by this study. The multi-ingredient strategy in performance supplementation certainly appears logical and at times empirically justified, but the current literature on MIPS does not provide adequate practical information that can properly guide athletes in making evidence-based decisions about how and if MIPS should be consumed.

Strictly within the limits of the present investigation, the overall findings fail to support the benefits of the experimental MIPS in conjunction with a six-week periodized RT program. As an executive summary of findings, supplementation of the experimental MIPS compound did not augment total weekly and overall volume load which likely explains the absence of detectable effects on training adaptations. The body of evidence regarding MIPS use are highly mixed. Given the evidential ergogenic benefits of key individual ingredients namely, creatine and beta-alanine, it is possible that the present investigational MIPS may elicit an effect on performance and/or body composition under different circumstances or populations compared to those applied in the current investigation. Due to the varying combinations of ingredients within the multitude of MIPS compounds, it remains difficult to ascertain superior compounds or optimum combinations and dosages of each ingredient. In the case of examining multi-ingredient supplements, a study design that incorporates individual and combined uses of the products of interest would be integral to enhancing the current understanding of the interactions among individual substances and the practical use of MIPS in sport and exercise. Accordingly, a paradigm shift in MIPS research away from a simple between-group comparison of a MIPS product vs. placebo and towards examining interactions and synergies among single-ingredient supplements would be warranted. As a follow up to the current investigation, it is evident considering the highlighted limitations in MIPS research that a step in reverse, so to speak, is strongly recommended to better justify the combined use of the various ingredients.

## Figures and Tables

**Table 1:** Between-group comparison of descriptive baseline measures.

Descriptive Measures	MIPS (*n* = 15) (7m, 8f)	PLA (*n* = 15) (7m, 8f)
Age (years)	21.9 ± 1.8	23.3 ± 1.8
Total Body Mass (kg)	63.3 ± 10.8	66.9 ± 12.0
Lean Mass (kg)	44.9 ± 9.0	49.0 ± 10.8
Fat Mass (kg)	15.9 ± 3.9	15.3 ± 5.4
Body Fat Percentage (%)	25.3 ± 5.2	22.8 ± 7.6
Height (cm)	165.8 ± 8.2	168.3 ± 9.2
Upper Body Strength (kg·kg TBM^−1^)	1.0 ± 0.4	1.0 ± 0.4
Lower Body Strength (kg·kg TBM^−1^)	1.4 ± 0.6	1.3 ± 0.4

Data presented as mean ± SD.

**Table 2:** Body composition changes across 12 weeks of resistance training in the multi-ingredient performance supplement (MIPS) and placebo (PLA) groups.

Variable	Group	PRE	POST	Time Effect (*p*-Value)
Total Body Mass (kg)	MIPS	63.3 ± 10.8	65.1 ± 11.2	<0.001
	PLA	66.9 ± 12.0	68.6 ± 12.7	<0.001
Lean Mass (kg)	MIPS	44.9 ± 9.0	47.2 ± 9.6	<0.001
	PLA	49.0 ± 10.8	50.6 ± 11.4	<0.001
Fat Mass (kg)	MIPS	15.9 ± 3.9	15.5 ± 4.0	=0.07
	PLA	15.3 ± 5.4	15.5 ± 6.0	=0.50

Data presented as mean ± SD.

**Table 3:** Percent change in strength variables from pre- to post-training in MIPS vs. PLA treatments, and qualitative inferences (QI) for the effects of MIPS on each variable.

Variable	MIPS (Mean ± SD)	PLA (Mean ± SD)	Mean Difference (MIPS − PLA)	*p*-Value	QI for Effect Magnitude (Mean Difference ± 90% CL)
∆ Squat 1RM (%)	26.8 ± 24.1	31.4 ± 25.6	−4.6	0.62	Unclear results (−4.6 ± 15.6)
∆ Bench 1RM (%)	20.5 ± 13.9	14.3 ± 12.4	6.1	0.21	Possibly beneficial (6.1 ± 8.1)
∆ DL 1RM (%)	27.6 ± 21.2	20.4 ± 15.4	7.3	0.29	Possibly beneficial (7.3 ± 11.5)
∆ Squat/LM (%)	20.9 ± 23.6	27.4 ± 23.7	−6.5	0.46	Unclear results (−6.5 ± 13.8)
∆ Bench/LM (%)	14.8 ± 13.0	11.0 ± 12.7	3.7	0.43	Possibly beneficial (3.7 ± 7.9)
∆ DL/LM (%)	21.6 ± 20.1	16.7 ± 14.4	4.8	0.46	Possibly beneficial (4.8 ± 10.9)

CL = Confidence Limit, LM = Lean Mass.

**Table 4:** Changes in lower body muscular power across 12 week of resistance training in MIPS and PLA.

Variable	Group	PRE	POST	Time Effect (*p*-Value)
70% PWR_AVG_ (W)	MIPS	416.6 ± 148.5	478.6 ± 151.1	<0.001
	PLA	431.2 ± 166.4	500.7 ± 162.0	<0.001
70% PWR_PEAK_ (W)	MIPS	901.2 ± 356.7	1129.2 ± 477.9	=0.001
	PLA	1031.1 ± 433.8	1211.2 ± 392.0	<0.001
80% PWR_AVG_ (W)	MIPS	416.6 ± 136.0	500.4 ± 161.1	<0.001
	PLA	439.1 ± 147.1	490.5 ± 153.9	=0.006
80% PWR_PEAK_ (W)	MIPS	963.1 ± 419.1	1216.3 ± 508.3	=0.002
	PLA	1060.3 ± 378.6	1241.9 ± 355.9	=0.002

70% and 80% PWR_PEAK_ = peak power during maximal effort squat using 70% and 80% one-repetition maximum (RM) load, respectively. 70% and 80% PWR_AVG_ = average power during maximal effort squat using 70% and 80% 1RM load, respectively. PRE = pre-training measurement, POST = post-training measurement. Data presented as mean ± SD.
